# A novel PCR-based method for high throughput prokaryotic expression of antimicrobial peptide genes

**DOI:** 10.1186/1472-6750-12-10

**Published:** 2012-03-23

**Authors:** Tao Ke, Su Liang, Jin Huang, Han Mao, Jibao Chen, Caihua Dong, Junyan Huang, Shengyi Liu, Jianxiong Kang, Dongqi Liu, Xiangdong Ma

**Affiliations:** 1 Department of Life Science and Technology, Nanyang Normal University, Wolong Road, Nanyang 473061, China; 2 Key Laboratory of Biology and Genetic Improvement of Oil Crops, Ministry of Agriculture, Oil Crops Research Institute, Chinese Academy of Agricultural Sciences, No.2 Xudong Second Road, Wuhan 430062, China; 3 Hubei Key Laboratory of Industrial Biotechnology, College of Life Science, Hubei University, Wuhan 430062, China; 4 School of Environmental Science and Engineering, Huazhong University of Science and Technology, Wuhan 430074, China

**Keywords:** antimicrobial peptide, high throughput, Npro, prokaryotic expression

## Abstract

**Background:**

To facilitate the screening of large quantities of new antimicrobial peptides (AMPs), we describe a cost-effective method for high throughput prokaryotic expression of AMPs. EDDIE, an autoproteolytic mutant of the N-terminal autoprotease, Npro, from classical swine fever virus, was selected as a fusion protein partner. The expression system was used for high-level expression of six antimicrobial peptides with different sizes: Bombinin-like peptide 7, Temporin G, hexapeptide, Combi-1, human Histatin 9, and human Histatin 6. These expressed AMPs were purified and evaluated for antimicrobial activity.

**Results:**

Two or four primers were used to synthesize each AMP gene in a single step PCR. Each synthetic gene was then cloned into the pET30a/His-EDDIE-GFP vector via an *in vivo *recombination strategy. Each AMP was then expressed as an Npro fusion protein in *Escherichia coli*. The expressed fusion proteins existed as inclusion bodies in the cytoplasm and the expression levels of the six AMPs reached up to 40% of the total cell protein content. On *in vitro *refolding, the fusion AMPs was released from the C-terminal end of the autoprotease by self-cleavage, leaving AMPs with an authentic N terminus. The released fusion partner was easily purified by Ni-NTA chromatography. All recombinant AMPs displayed expected antimicrobial activity against *E. coli*, *Micrococcus *luteus and *S. cerevisia*.

**Conclusions:**

The method described in this report allows the fast synthesis of genes that are optimized for over-expression in *E. coli *and for the production of sufficiently large amounts of peptides for functional and structural characterization. The Npro partner system, without the need for chemical or enzymatic removal of the fusion tag, is a low-cost, efficient way of producing AMPs for characterization. The cloning method, combined with bioinformatic analyses from genome and EST sequence data, will also be useful for screening new AMPs. Plasmid pET30a/His-EDDIE-GFP also provides green/white colony selection for high-throughput recombinant AMP cloning.

## Background

Antimicrobial peptides are widely distributed in nature and play a critical role in the innate immunity of host defense systems. They act with broad spectrum and, hence, are promising candidates for therapeutic and industrial application [1-5]. For research studies and clinical trials, large quantities of these peptides are needed [6]. The number of described AMPs has increased over recent decades [7]; however, the recent generation of huge amounts of genomic, proteomic and EST (Expressed Sequence Tag) data enables novel strategies for the discovery of new candidate AMPs [8-10]. *In silico *methods based on bioinformatic analyses, combined with experimental screening techniques have been developed to screen and identify new AMP genes from huge "-omics" data sets [11,12]. Belarmino *et al. *screened 237,954 ESTs of sugarcane using a computational approach and successfully identified 17 new defensin isoforms [13]. Following *in silico *prediction, however, there is a requirement for a high throughput genome-scale DNA cloning and expression system to enable the antimicrobial activities of putative AMPs to be characterized [9,14,15].

Expression of fusion proteins that form inclusion bodies has several advantages that can overcome major barriers of AMP expression in *E. coli*: high rates of expression, easy collection by centrifugation, protection from proteolysis and the avoidance of intrinsic AMP antimicrobial activity against host cells [16,17]. However, recombinant AMPs expressed in *E. coli *often include 1-2 non-native amino acid residuals at the N terminus of the target protein due to a specific linker sequence recognized by endoproteases or chemical agents, typically located between the native protein sequence and the tag [18]. These non-authentic N termini of AMPs often alter their characteristics. It is, therefore, very important to develop a new strategy for authentic recombinant AMP expression. The N-terminal autoprotease, Npro, of classical swine fever virus (CSFV) cleaves itself between the C-terminal Cys168 and position 169 [19], which represent the authentic N-terminal amino acid of the target protein. It is also a relatively hydrophobic protein that tends to form insoluble aggregates on refolding, preventing autoproteolysis [20]. In this approach, the target protein is fused to the C-terminus of Npro and is expressed in inclusion bodies. After inclusion bodies are isolated, an *in vitro *refolding step is necessary to induce autoproteolysis, and render the AMPs biologically active [21]. Importantly, cleavage does not need to be initiated by the use of reducing agents or by temperature and pH shifts. EDDIE, a mutant of Npro, shows improved solubility and faster refolding and cleavage than wild-type Npro [16]. In our previous work, the fusion protein EDDIE-CAD was successfully expressed in *E. coli *after codon optimization and the purified recombinant mature Cecropin AD (CAD) was fully bioactive [22].

**Figure 1:**
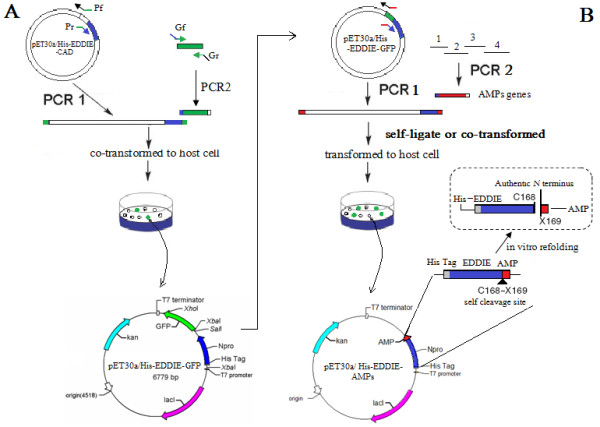
**Schematic representation of the Construction of (A) pET30a/His-EDDIE-GFP and (B) pET30a/His-EDDIE-AMPs vectors**. **(A) **pET30a/His-EDDIE-CAD was used to constructed pET30a/His-EDDIE-GFP. This plasmid was derived from pET30a and uses a T7-inducible promoter with lac operator, contains the low-copy pBR322 origin of replication, and encodes the kanamycin resistance gene (kanR) and the lac repressor gene (lacI). The GFP gene was inserted at the Sal I site and replaced CAD gene of pET30a/His-EDDIE-CAD, which at downstream positions of the EDDIE gene, give rise to the vector pET30a/His-EDDIE-GFP for expression of AMPs in E. coli. **(B) **The AMPs genes were inserted downstream of the carrier protein using overlap primer (arrows) at 168 site. The separate of AMPs and carrier partner between self-cleavage sites while in vitro refolding is shown in the square box, while the self-cleavage site is indicated by an arrow.

The *in vitro *cloning of DNA molecules traditionally uses PCR or site-specific restriction endonucleases to generate linear DNA inserts with defined termini and requires DNA ligase to covalently join these inserts to vectors with the corresponding ends [23]. However, restriction endonuclease sites often introduce a few amino acids to the N-terminus of mature peptides, and it is very difficult to analyze recombinant clones when the insert DNA fragment is very short. For high throughput subcloning of short peptide genes, we constructed the vector, pET30a/His-EDDIE-GFP (Figure 1A), and used a seamless enzyme-free cloning method for high level expression of AMPs in *E. coli*, This method allows one-step assembly of DNA fragments *in vivo *via homologous recombination in *E. coli *[24]. For high level expression, codon usages of synthesized AMP genes were optimized according to the host strains. For efficient analysis of short insert DNA fragments, the recombinants were easily screened by GFP green/white colony selection. In this article, our approach was validated using 6 target AMPs of variable length.

## Results

### Construction of his-EDDIE-GFP fusion expression plasmid

**Figure 2:**
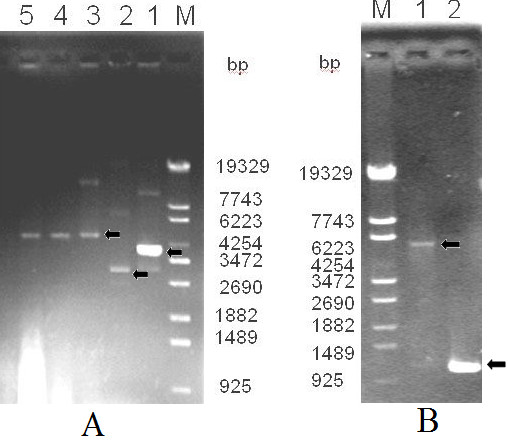
**Agarose gel electrophoresis of the PCR products of the GFP gene and of plasmid pET30a/His-EDDIE-GFP**. **(A) **Lane M: molecular weight marker; Lane 1: plasmid of pET30a/His-EDDIE-CAD; Lane 2: plasmid of pET30a; Lane 3-5: plasmid of pET30a/His-EDDIE-GFP; **(B) **Lane M: molecular weight marker; Lane 1: PCR products of linearized plasmid pET30a/His-EDDIE-GFP; Lane 2: PCR products of GFP genes used pET30a/His-EDDIE-GFP as template.

The construction of the pET30a/His-EDDIE-GFP vector was based on pET30a/His- EDDIE-CAD. After PCR amplification, the GFP fragment was successfully subcloned into pET30a/His-EDDIE-CAD. Colonies showing green fluorescence were picked and PCR amplification of the GFP gene followed by gel electrophoresis showed that the GFP fragment had been successfully inserted into the expression vector (Figure 2A, B). DNA sequencing validated that the GFP gene was inserted downstream of the EDDIE gene.

### Construction of the his-EDDIE-AMP fusion expression plasmid

**Figure 3:**
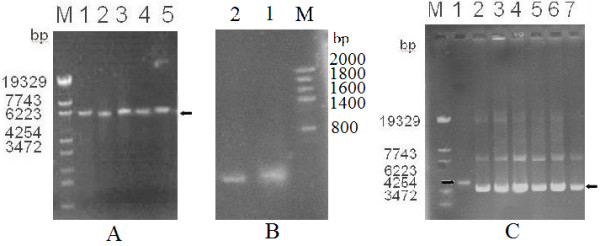
**Gel analysis of six recombinant pET30a/His-EDDIE-AMP expression vectors**. **(A) **Lane M: molecular weight markers; Lane 1-4: PCR product of four recombinant expression vector pET-His-EDDIE-AMPs; Lane 5: PCR product of vector pET-His-EDDIE-GFP; **(B) **Lane M: molecular mass makers; Lane 1-2: PCR product of Histatin6 and BLP-7 genes; **(C) **Lane M: molecular mass makers; Lane 1: plasmid of pET30a/His-EDDIE-GFP; Lane 2-7: plasmids of six pET30a/His-EDDIE-AMPs;.

To clone AMP genes in a high-throughput manner, we performed a green/white colony screen using the GFP in pET30a/His-EDDIE-GFP. After a target gene is cloned into the vector by a one-step PCR technique, the GFP gene is destroyed; therefore, green fluorescence indicates non-linearized parental vector. Four AMP genes, Temporin G, hexapeptide, Combi-1 and Histatin 9 were amplified and cloned downstream of EDDIE using a one-step PCR process, and then transformed into *E. coli *cells (Figure 3A). BLP-7 and Histatin 6 genes were assembled using four primers in one PCR reaction, respectively (Figure 3B). pET30a/His-EDDIE-GFP was replicated at the same time. The two PCR products were then transformed into *E. coli *cells together. The transformants were screened under ultraviolet light, and the white colonies were further screened by PCR verification and sequencing. The recombinant pET30a/His-EDDIE-AMP plasmid is smaller than the parental pET30a/His-EDDIE-GFP plasmid, which makes it very easy to distinguish the two plasmids by agarose gel electrophoresis (Figure 3C).

### Expression and purification of fusion proteins

**Figure 4:**
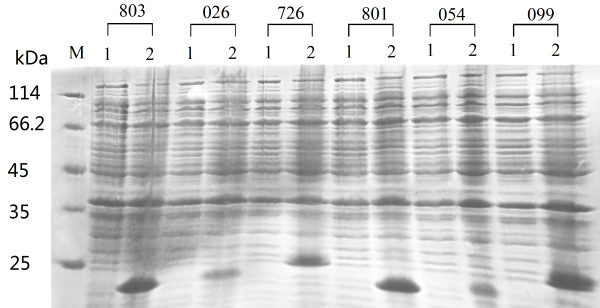
**SDS-PAGE analysis of recombinant His-EDDIE-AMPs expressed in *E. coli *BL21 (DE3)**. Lane M: the molecular weight markers; Lane 1: crude cells extracts of uninduced *E. coli *BL21 containing pET-His-EDDIE-AMP; Lane 2: crude cells extracts after 5 h past the induction with IPTG of *E. coli *BL21 containing pET-His-EDDIE-AMP; The molecular weights of the new protein components agree well with those predicted for the fusion proteins.

*E. coli *BL21 (DE3) cells harboring pET30a/His-EDDIE-AMP were induced by IPTG, and the expression of His-EDDIE-AMP proteins was analyzed by SDS-PAGE (Figure 4). Fusion proteins of 20 kDa represented the majority of the insoluble components in cell lysates. The recombinant His-EDDIE-AMPs were estimated to constitute about 40% of the total protein present in cells. The yield of inclusion bodies was quite high due to the properties of the fusion partner (high content of hydrophobic residues).

### Refolding and activity analysis

**Figure 5:**
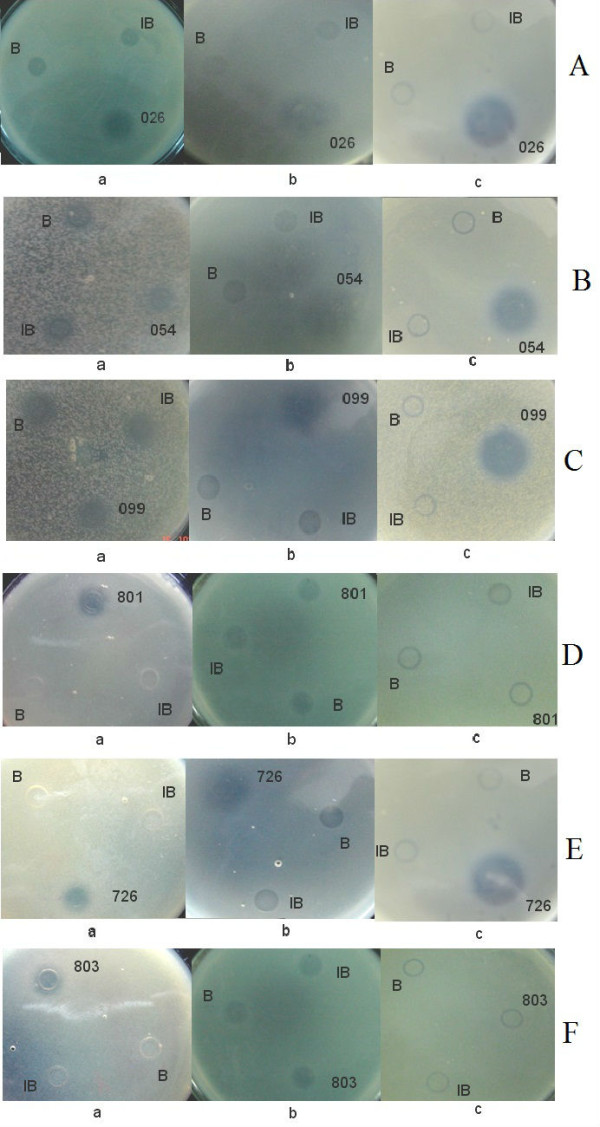
**Detection of the antibacterial activities of candidate antimicrobial peptides against *E. coli *and *M. luteus***. B: refolding buffer; IB: Inclusion bodies of His-EDDIE-AMPs; a: antimicrobial activities assay against *S. cerevisiae*; b: antimicrobial activities assay against *E. coli*; c: antimicrobial activities assay against *M. luteus*; **(A) **026, Hexapeptide; **(B) **054, BLP-7; **(C) **099, Temporin G; **(D) **801, human Histatin 6; **(E) **726, Combi-1; **(F) **803, human Histatin 9.

Purified His-EDDIE-AMP inclusion bodies were diluted in optimized refolding buffer and incubated to enable self-cleavage to occur. To examine the antimicrobial activity of six recombinant AMPs, the purified supernatants were assayed using a radial diffusion assay. As shown in Figure 5, there were large halos around the six AMPs, indicating that all six AMPs had specific bactericidal activities against *E. coli *ATCC2592, *M. luteus or S. cerevisiae*. No inhibition zones were seen around the negative control spots. The recombinant AMPs were clearly bioactive and very effective in killing these sensitive strains.

## Discussion

We have described a new method for cloning and expressing AMP genes. The desired AMPs are amplified using ORF-specific primers with flanking sequences identical to the two ends of a linearized vector. The PCR product and the linearized vector are then cotransformed into *E. coli *cells, where the ORF is incorporated into the vector *in vivo*. Short AMPs can even directly incorporate into the linearized vector through self-ligation. Unlike conventional methods that use restriction enzymes or site-specific recombinases, PCR products could be seamlessly assembled without the need for specific sequences for ligation or site-specific recombination [25]. This system is more efficient than cloning methods requiring ligase [26,27] and will be useful for standard DNA cloning and for constructing genome-scale clone resources that facilitate functional analysis [23].

Most AMP genes are very small (100-200 bases) and it is difficult to identify recombinant plasmids containing these genes. We, therefore, incorporated the GFP reporter gene into the expression vector to enable convenient recombinant colony picking.

AMPs expressed as Npro fusions are deposited as insoluble proteins in inclusion bodies. This greatly increases yield due to resistance to proteolytic degradation. During refolding *in vitro*, EDDIE self-cleaved at the specific site and the six target peptides were released. Our construct also encodes the (His)_6 _affinity tag, preceding the coding regions of EDDIE, to enable fast and straightforward purification using Ni^2+^-chelating affinity chromatography after refolding. The target peptides could be easily purified to homogeneity.

These results validated this high throughput AMP subcloning method. Using this method, we successfully cloned 40 peptides into the expression vector and identified about 20 new AMPs from *Brassica napus *cDNA libraries that showed antimicrobial activities (data not shown). This approach, combined with bioinformatic analyses of genome and EST sequence data, will be useful in screening for new AMPs.

## Conclusions

In conclusion, this is a simple, reliable, automated, robust, flexible and low-cost expression cloning approach in which PCR fragments are fused into an expression vector without unwanted amino acids. This strategy advances the methodologies available for AMP prokaryote expression. The main barriers to AMP expression, such as, codon preference, the intrinsic antimicrobial activity of AMPs to host cells, and inefficient production of AMPs owing to proteolytic degradation, are overcome by this expression system.

## Methods

### Materials

*E. coli *XL-GOLD (Stratagene, USA) was used as the host for subcloning and plasmid amplification. *E. coli *BL21 (DE3) was used as the host for expression of recombinant protein. *E. coli *ATCC2592 and *M. luteus *ACCC11001 were used as indicators in the antimicrobial assay of the six antimicrobial peptides. pET30a (Novagen, Madison, WI, USA) was used as a vector construction and recombinant protein expression plasmid. Restriction enzymes *Nde*I and *Sal*I were purchased from Takara (Dalian, China).

### Construction of the pET30a/His-EDDIE-GFP expression vector

The plasmids containing the Npro mutant, EDDIE, were constructed according to Zhang *et al. *[22]. To enable green/white screening of recombinant clones (green colonies indicate the presence of uncut parental vector), the PCR primers Gf and Gr were designed according to the reported DNA sequence of GFP (GenBank accession no. ABN41558), with an added 18 bases flanking the sequences complementary to the two ends of linearized vector (Table 1). The pET30a/His-EDDIE-CAD vector was amplified using primers Pf and Pr (25 cycles of 95°C for 10 s, 65°C for 30 s, and 72°C for 6 min using Pyrobest DNA polymerase; Takara Bio Inc., Shiga, Japan), and the linear PCR product, with 18 bases at each end homologous to GFP, was digested by *DpnI *and purified. The plasmid was then generated by the seamless enzyme free cloning method [24], and was named pET30a/His-EDDIE-GFP (Figure 1). 5 μL of purified PCR product (100-300 ng) and 1 μL (50 ng) of the appropriately linearized vector were mixed and transformed into 50 μL of *E. coli *XL-GOLD chemically competent cells by heat shock and then plated on selection plates (containing 50 μg/mL kanamycin). The recombinant colonies were easily selected by visualizing GFP fluorescence under ultraviolet light.

**Table 1:** Primers for vector construction

Primer name	Sequence(5'-3')
Pf	GACACAGCGAACGGCGCAGCTGGTCACCCACAGCGGGCAAT

Pr	GAGCTGTACAAGTGAAAGCTTGCGGCCGCACTCGAGCAC

Gf	GCCGTTCGCTGTGTCGCACAAT

Gr	TCACTTGTACAGCTCGTCCATGCCAT

### Construction of the AMP expression vector with EDDIE as a fusion partner

Six AMPs were selected from the AMP database [28] (Table 2). The AMP sequences were optimized according to *E. coli *codon usage. For short AMPs, the sense and antisense primers contained the reported DNA sequence and 18 bases of overlap with each other. Their 3' ends also contained the EDDIE C-terminal sequences and the ends of MCS of the vector, respectively. For longer AMPs, BLP-7 and Histatin 6, genes were assembled using 4 primers in a one-step PCR reaction. Vector was amplified with the PCR primers, backboneF and backboneR, which were designed according to the EDDIE C-terminal sequences and the ends of MCS of the vector, respectively (Table 3). The pET30a/His-EDDIE-GFP vector was amplified with the primers, and the linearized vector was purified and digested with Dpn*I*. The PCR reaction was carried out for 25 cycles, each cycle consisting of 30 s at 94°C, 30 s at 62°C, and 7 min at 72°C. The PCR products covalently join to vectors with the corresponding ends *in vivo *when transformed into *E. coli*. White colonies were picked, and then sequenced to ensure that the coding sequence was correct. The resulting plasmids were named pET30a/His-EDDIE-AMPs, respectively (Figure 1B).

**Table 2:** Antimicrobial peptides expressed in this research

AMP database No.	Origin	Amino acid sequences of mature peptide	Length	Anti characteristics
AP00054	Bombinin-like peptide 7, BLP-7	GIGGALLSAGKSALKGLAKGLAEHFAN	27	Gram + & Gram-

AP00099	Temporin G	FFPVIGRILNGIL	13	Gram + & Gram-

AP00026	Hexapeptide, LfcinB6	RRWQWR	6	Gram + & Gram-, Virus, Fungi, Cancer cells

AP00726	Combi-1	RRWWRF	6	Gram + & Gram-, Fungi

AP00803	human Histatin 9	RKFHEKHHSHRGYR	14	Fungi

AP00801	human Histatin 6	DSHAKRHHGYKRKFHEKHHSHRGYR	25	Fungi

**Table 3:** Antimicrobial peptides expressed in this research

AMPs	Name	Oligomer sequence (5'-3')
Hexapeptide	026 F	TTAACGCCATTGCCAGCGACGGCAGCTGGTCACCCACAG
	
	026R	CGTCGCTGGCAATGGCGTTAAAAGCTTGCGGCCGC

Combi-1	726 F	TTAGAAACGCCACCAACGACGGCAGCTGGTCACCCACAG
	
	726R	CGTCGTTGGTGGCGTTTCTAAAAGCTTGCGGCCGC

Temporin G	099 F	CAGAATGCGGCCAATGACCGGAAAAAAGCAGCTGGTCACCCACAG
	
	099R	ATTGGCCGCATTCTGAATGGCATCCTGTAAAAGCTTGCGGCCGC

human Histatin 9	803 F	TGGCTGTGGTGTTTCTCATGGAATTTACGGCAGCTGGTCACCCACAG
	
	803R	AGAAACACCACAGCCATCGTGGGTATCGTTAAAAGCTTGCGGCCGC

BLP-7	054-1	CTGTGGGTGACCAGCTGCGGCATTG
	
	054-2	GGGCGCTTTTTCCCGCGCTCAGCAGCGCGCCTCCAATGCCGCAGCTGGTC
	
	054-3	GCGGGAAAAAGCGCCCTGAAAGGCCTGGCGAAAGGCTTGGCGGAACATTT
	
	054-4	GCGGCCGCAAGCTTTTAATTCGCAAAATGTTCCGCCAAGCCTT

human Histatin 6	801-1	CTGTGGGTGACCAGCTGC
	
	801-2	CTTATAGCCGTGATGACGCTTCGCATGGCTATCGCAGCTGGTCACCCACA
	
	801-3	AAGCGTCATCACGGCTATAAGCGCAAGTTTCACGAAAAACACCACAGCCA
	
	801-4	GCGGCCGCAAGCTTTTAACGATAACCACGATGGCTGTGGTGTTTTTCGT

	backbone eF	TGAGATCCGGCTGCTAACAAAGCCC

	backbone eR	GCAGCTGGTCACCCACAGCG

### Expression and purification of fusion protein

The pET30a/His-EDDIE-AMP plasmids were transformed into the expression host, *E. coli *BL21 (DE3) (Novagen, Madison, WI, USA). One colony was used to inoculate 50 mL LB (1% Bacto-tryptone, 0.5% yeast extract, and 8 mM NaCl) medium supplemented with 50 μg/mL kanamycin, and grown overnight in a 37°C in a shaking incubator. The fully grown culture was mixed with 1 L LB medium with the same antibiotics the next morning. The culture was grown at 25°C, and IPTG was added to a final concentration of 1 mM when the OD_600 _reached 0.5. The culture was harvested 5 h later and the cells were washed and resuspended in 30 mL PBS buffer (NaCl 137 mM, KCl 2.7 mM, Na_2_HPO_4 _4.3 mM, KH_2_PO_4 _1.4 mM, pH 7.2-7.4). The cells were lysed by freeze-thaw and the DNA was fragmented by ultrasonication. The insoluble inclusion bodies were isolated by 14,000 × g centrifugation for 30 min in 4°C. The pellet was washed three times with washing buffer (10 mM Tris/HCl, pH 7.6; 200 mM NaCl, 2 mM 2-mercaptoethanol, and 1% Triton X-100) and then solubilized in denaturing buffer (8 M urea; 20 mM Tris-HCl, pH 7.6; and 5 mM 2-mercaptoethanol).

### Refolding and activity analysis

Purified His-EDDIE-AMPs inclusion bodies were refolded by rapid 1:50 dilution in optimized refolding buffer (500 mM NaCl, 20 mM Tris, 2 mM EDTA, 5% glycerol, 10 mM DTT, 0.01% Tween-20, pH 7.5) and incubated at an appropriate temperature without stirring. During refolding, EDDIE restored its correct conformation and self-cleaved at the specific site, releasing AMPs from the fusion bodies. Renatured protein solution was then clarified by 15,000 × g centrifugation for 30 min in 4°C. Then the insoluble sample was removed by filtering through 0.45 μm membrane and AMPs were left in the supernatant. The supernatants were applied to a Ni-NTA His-bind column for purification.

Standard SDS-PAGE (12% gel) was applied to assay fusion proteins. Band density was analyzed using a GEL-DOC 2000 gel documentation system (BIO-Rad, Hemel Hempstead, UK) and Quantity One software, version 4.4.0 was used to determine the fraction of target protein. EDDIE protein was quantified using a BCA protein assay kit (Pierce, Rockford, IL, USA). Antimicrobial activity of recombinant AMPs was detected using a radial diffusion assay [29]. Briefly, *E. coli *ATCC2592, *M. luteus *ACCC11001 and *S. cerevisiae *were grown to the mid-logarithmic phase and washed. Approximately 2 × 10^6 ^cfu/mL bacteria were incorporated into a thin (1.2 mm) agarose underlay gel that contained 1% (wt/vol) agarose. Holes of 3.5 mm diameter were punched into the solidified agarose and these were filled with 100 μL of AMP sample. After the plates were incubated for 12 h at 37°C, the diameter of the clear zone surrounding each well was measured to evaluate the antimicrobial activity. Refolding buffer and inclusion bodies were used as negative controls. The above assays were performed in triplicate.

## Competing interests

The authors declare that they have no competing interests.

## Authors' contributions

TK and XDM made substantial contributions to conception and design. TK and SL carried out the experiments and wrote the manuscript. JBC and JXK participated in drafted the manuscript. JH, JYH and DQL participated in the experiments. HM, CHD and SYL participated in reviewing the manuscript and given final approval of the version to be published. All authors read and approved the final manuscript.
